# Evaluation of DNA markers for molecular identification of three *Piper* species from Brazilian Atlantic Rainforest

**DOI:** 10.1371/journal.pone.0239056

**Published:** 2020-10-19

**Authors:** Anary P. M. Egydio Brandão, Lydia F. Yamaguchi, Eric J. Tepe, Antonio Salatino, Massuo J. Kato

**Affiliations:** 1 Department of Botany, University of São Paulo, São Paulo, São Paulo, Brazil; 2 Institute of Chemistry, University of São Paulo, São Paulo, São Paulo, Brazil; 3 Department of Biological Sciences, University of Cincinnati, Cincinnati, Ohio, United States of America; Institute for Biological Research "S. Stanković", University of Belgrade, SERBIA

## Abstract

*Piper* is one of two large genera in the Piperaceae, and with ca. 2600 species, is one of the largest plant genera in the world. Species delimitation and evaluation of genetic diversity among populations are important requisites for conservation and adequate exploitation of economically important species. DNA barcoding has been used as a powerful tool and a practical method for species characterization and delimitation. The present work aims to evaluate molecular markers for barcoding three *Piper* species native to Brazil: *P*. *gaudichaudianum* (“jaborandi” or “pariparoba”), *P*. *malacophyllum* (“pariparoba-murta”) and *P*. *regnellii* (“caapeba” or “pariparoba”). A reference DNA barcode library was developed using sequences of three candidate regions: ITS2, *trnH-psbA* and *rbcL*. Transferability of the microsatellite (SSR) primers Psol 3, Psol 6 and Psol 10, designed originally for *Piper solmsianum*, to the three *Piper* species was also evaluated. The discriminatory power of the markers was based on the determination of inter- and intraspecific distances, phylogenetic reconstruction, and clustering analysis, as well as BLASTn comparison. Sequences of ITS2 enabled efficient species identification by means of the BLASTn procedure. Based on these sequences, intraspecific divergence was lower than interspecific variation. Maximum Parsimony analyses based on ITS2 sequences provided three resolved clades, each corresponding to one of the three analysed species. Sequences of *trnH-psbA* and *rbcL* had lower discriminatory value. Analyses combining sequences of these regions were less effective toward the attainment of resolved and strongly supported clades of all species. In summary, robustly supported clades of *P*. *regnellii* were obtained in most of the analyses, based either on isolated or combined sequences. The SSRs primers Psol 3, Psol 6 and Psol 10 were shown to be transferable to *P*. *gaudichaudianum* and *P*. *regnellii*, but not to *P*. *malacophyllum*. Preliminary cluster analyses based on the polymorphism of the amplified products suggested that Psol 3 has lower potential than Psol 6 and Psol 10 for discrimination of *Piper* species.

## Introduction

Family Piperaceae is one of the largest groups of basal angiosperms, with species widely distributed in tropical and subtropical regions of both New and Old worlds. *Peperomia* Ruiz & Pav. and *Piper* L. are the genera in the Piperaceae with the highest number of species, with ca. 2,000 and 2,600 taxa, respectively [1–3]. Several species of *Piper* are frequently used as spices, as medicines or as ornamentals. *Piper nigrum* L. (black pepper) is one the most consumed spices in the world, and *P*. *methysticum* G. Forst. (“kava-kava”) is widely used to treat anxiety disorders [4].

*Piper malacophyllum* (C.Presl) C.DC. and *P*. *regnellii* (Miq.) C.DC. are native to Brazil and *P*. *gaudichaudianum* Kunth occurs more widely in Latin America. No genomic data have been published for either *P*. *regnellii* or *P*. *malacophyllum*. The three species are used in popular medicine for treatment of many diseases. For example, *P*. *regnellii* is used as anesthetic and anti-inflammatory and *P*. *gaudichaudianum*, is used to treat toothaches [5]. Extracts of these species have revealed activity against human and plant fungal pathogens [6–8]. On the other hand, frauds, misuses, and misidentifications of plant names are common in the market of medicinal plants. In addition, vernacular names are often used to refer to several species, due to similarities in the morphology of these plants and their traditional uses. Thus, “pariparoba” may apply to *P*. *regnellii*, *P*. *gaudichaudianum*, *P*. *malacophyllum*, *P*. *umbellatum* L. and other species. The development of molecular tools may contribute to the correct identification of these useful species.

DNA barcoding is a rapid and reliable procedures aimed at the accurate identification and delimitation of species [9]. As part of the DNA barcoding protocol, sequences of unidentified samples are compared against a database of know-DNA sequences [10–11]. Certain properties of DNA markers are essential for barcoding purposes: a) high interspecific polymorphism, b) low or non-existing intraspecific divergence, c) existence of conserved flanking regions, allowing for the design of universal primers [12–14]. The mitochondrial gene *Cox* I (or COI) has been universally employed for barcoding animal species [15]; however, a single marker that is useful across all plants has not yet been identified. Nevertheless, a comprehensive review [16] revealed multiple markers that could be used as DNA barcodes within a given plant group. The CBOL-Consortium for the Barcode of Life has recommended the plastid gene ribulose-bisphosphate carboxylase (*rbcL*) [17], because of its universality, sequence quality and differentiation power [14,18]. Although *rbcL* has been useful for distinction between species [19], its usefulness is usually restricted to the levels of family and genus. For this reason, markers with high degree of variation have been recommended [10], such as the nuclear Internal Transcribed Spacer (ITS) and the plastid *trnH-psbA* intergenic spacer [12]. The plastid DNA regions *rbcL*, *matK* and *trnH-psbA* have been used for DNA barcode of Pteridophytes [20], Cycadales [21], Fabaceae [22], in addition to several other taxa.

Previous attempts at barcoding Old World *Piper* species included the use of chloroplast regions *matK*, *rbcL*, *rpoC1* and *trnH-psbA*, as well as the nuclear ITS2 markers [23–24]. The barcoding analysis of thirty-six *Piper* species from Thailand was carried out using *matK*, *rbcL* and *trnH-psbA* [25]. In these studies, most species could be accurately identified using the DNA barcodes.

Microsatellites, also known as SSRs (Simple Sequence Repeats), are simple and short DNA sequences, consisting of two to six nucleotides repeated in tandem. Their polymorphism results from variation of the number of sequence repeats [26]. Due to their specificity, codominant inheritance, high abundance in all eukaryotic genomes, high polymorphism, good reproducibility, versatility and ease of use, SSRs have become valuable markers for a wide range of applications, including barcoding, mapping, marker-assisted breeding [27], and evaluation of plant genetic variability [28]. Arguments have been put forward regarding the value of SSRs for authentication and identification of medicinal plants [29], spices and other food plants [30–31]. For instance, nine SSR markers were used for the identification of Chinese samples of ginseng [31]. The use of sixty SSR markers showed high polymorphic levels among 192 *Capsicum* lines, allowing the assembling of five groups [30]. Additionally, integrated SSRs and sequences of chloroplast *rpo*C1, aimed at the identification of the ’Eglouvi’ lentil variety was also carried out [32]; the combination of molecular markers was used to detect adulterants at low cost, suggesting its use could be a valuable tool in forensic sciences and determination of food safety. Within *Piper*, SSRs were used to assess the genetic diversity among commercial genotypes of *P*. *nigrum* in Southern India [33], as well as the infraspecific genetic variation of *P*. *methysticum* [34] and *P*. *solmsianum* C. DC. [35]. Finally, information from SSR amplification profiles, along with morphological characters traditionally used in *Piper* taxonomy were used to identify the most efficient data set for species distinction [36].

In spite of the cost and time-consuming development of specific SSR primers [37], several works have shown that primers developed for one species can be used to amplify SSRs of phylogenetically related species. Primer transferability among species demonstrated that the regions flanking of microsatellites can be highly conserved [38–39], thus making it possible for primers developed for one species to produce useful data in other closely related species. Transferability of SSR primers, for example, have been verified for *Secale cereale* L., *Triticum aestivum* L. [37], *Ficus* [40], *Betula*, *Corylus* [39], *Lolium* [41], *Arachis* [42], many other species. Previous investigation about transferability of SSR markers to *Piper* revealed that markers designed for *P*. *solmsianum* provided the highest transferability rate among *Piper* species [36].

The present study aims to evaluate sequences of DNA regions toward barcoding of three *Piper* species native from Brazil: *P*. *gaudichaudianum* (“jaborandi”), *P*. *malacophyllum* (“pariparoba-murta”) and *P*. *regnellii* (“caapeba” or “pariparoba”). In addition, it is intended to evaluate the transferability of SSR primers, previously designed for *P*. *solmsianum* [35], to the three mentioned species and evaluate their efficacy for barcoding purposes.

## Material and methods

### Plant material

Fresh leaves from 20 specimens of *P*. *gaudichaudianum*, 20 of *P*. *regnellii* and 20 of *P*. *malacophyllum* were collected. Plant material was obtained from specimens cultivated at the garden of the Laboratory of Natural Product Chemistry, Institute of Chemistry, University of São Paulo (LQPN-USP), and from specimens growing in the Intervales State Park (State of São Paulo, Southeast Brazil) (Table 1). All plant material was identified by Dr. Elsie F. Guimarães, and voucher specimens were deposited in the Herbarium of the Research Institute of the Botanical Garden of Rio de Janeiro, Brazil (RB).

**Table 1:** Samples of *Piper* species used for analyses involving DNA regions and microsatellites (SSRs).

Species	Voucher	Sampling sites	N
*P*. *gaudichaudianum* Kunth	M. Kato K-1983	Parque Estadual Intervales, Ribeirão Grande—SP	10
*P*. *gaudichaudianum* Kunth	M. Kato K-031	USP, São Paulo—SP	10
*P*. *malacophyllum* (C.Presl) C.DC.	M. Kato K-448	Parque Estadual Intervales, Ribeirão Grande—SP	04
*P*. *regnellii* (Miq.) C.DC.	M. Kato K-1452	Parque Estadual Intervales, Ribeirão Grande—SP	10
*P*. *regnellii* (Miq.) C.DC.	M. Kato K-242	USP, São Paulo -SP	10

### DNA extraction

Genomic DNA was extracted using either the NucleoSpin Plant II kit (Macherey-Nagel-MN) or the Dneasy Plant Mini (Qiagen) kits, following the manufacturers’ protocols. The quality of the DNA obtained was evaluated by electrophoresis using TAE 1X buffer and 2% agarose gel containing GelRed (Amersham).

### Amplification and sequencing of DNA regions

Primers used for amplification of DNA regions are shown on Table 2. Polymerase chain reactions (PCRs) were performed with 50 μL reaction mixtures, containing 10X Taq reaction buffer, dNTPs in the final concentration of 0.2 mM, each primer at 10 μM, 1.25 U Taq Polymerase (Promega, USA), 10 μL of 1% PVP (polyvinylpolypyrrolidone), 20–50 ng of total DNA template and milliQ water to make up the volume of 50 μL. Thermal cycling conditions for ITS2 were: 1 min at 97°C, 40 cycles of 1 min at 97°C, 1 min at 48°C, 45 sec at 72°C and 7 min at 72°C. For *rbcL*, the following protocol was used: 5 min at 94°C, 30 cycles of 1 min at 94°C, 30 sec at 50°C, 1 min at 72°C and a final extension of 7 min at 72°C. The following protocol was used for *trnH-psbA*: 5 min at 94°C, 30 cycles of 30 sec at 94°C, 30 sec at 48°C, 30 sec at 72°C and a final extension of 5 min at 72°C.

**Table 2:** Primer sequences used for amplification of DNA regions of *Piper* species.

*Primers*	*Sequences*	*References*
**ITS2**		
IITS2-S2F	ATGCGATACTTGGTGTGAAT	[44]
ITS4	TCCTCCGCTTATTGATATGC	
***rbcL***		
*rbcL*a-F	ATGTCACCACAAACAGAGACTAAAGC	[10]
*rbcL*a-R	GTAAAATCAAGTCCACCRCG	
***trnH-psbA***		
*psbA*F	GTTATGCATGAACGTAATGCTC	[44]
*trnH*2	CGCGCATGGTGGATTCACAATCC	

Amplification was performed in a MyCycler Thermal Cycler (BioRad). Purification of the amplified products was performed by combining two enzymes: shrimp alkaline phosphatase (SAP) and exonuclease I (EXO). For each reaction, 8 μL of the amplified product and 2 μL of the ExoSAP-IT enzyme kit were used. The reaction was incubated in thermocycler for 1 h at 37°C and 15 min at 80°C.

Reactions for sequencing were prepared in 96-well plates with the BigDye^®^ Terminator kit version 3.1 (Applied Biosystems), according to the manufacturer’s protocol. Each reaction contained 3 μL of the amplified product, 4.7 μL of milliQ water, 0.30 μL of the primer and 2 μL of BigDye^®^ Terminator. The labeled products were precipitated with 60 μL of 70% isopropanol and centrifuged at 4000 rpm for 45 min. After discarding the supernatant, 170 μL of 70% ethanol was added. Centrifugation was done at 4000 rpm for 10 min, the supernatants were discarded, and the pellets dried in incubator at 37°C. The DNA was resuspended following standard procedures, and was denatured at 94°C for 2 min, followed by immediate cooling on ice. Sequencing was performed using an ABI Prism 3100 (Perkin Elmer) Genetic Analyzer. The forward and reverse sequences of the same sample were complemented, assembled, and aligned with ClustalW [43], followed by manual optimization with BioEdit. Sequences of each plant sample were organized into matrices for phylogenetic analysis.

### Amplification and analysis of SSR fragments

Development of the SSR primers (Table 3), Psol 3, Psol 6, and Psol 10 was described elsewhere [35]. Polymerase chain reactions (PCRs) were performed in 20 μL reaction mixtures, containing 10 mM Tris-HCl and 50 mM KCl buffer, 20–50 ng of total DNA template, 2 mM MgCl_2_, dNTPs at 0.25 μM, bovine serum albumin (BSA) at 0.5 μg.μL^−1^, forward primer (F) at 0.16 μM, reverse primer (R) at 0.2 μM, M13 primer IR700 or IR800 at 0.3 μM, and 1 U of *Taq* DNA polymerase. One of the primers of each of the six pairs contained a fluorophore (IRDye-700 or IRDye-800) for capillary electrophoretic detection. For PCR amplification, the following program of temperatures was used: 5 min at 94°C, 10 cycles of 1 min at 94°C, 1 min at 58°C, decreasing 1°C at each following cycle, 1 min at 72°C, 30 cycles of 40 sec at 94°C, 40 sec at 48°C, 1 min at 72°C, and a final extension of 10 min at 72°C [31].

**Table 3:** Characteristics of nuclear microsatellites (SSRs) used for *Piper* species.

Locus	Primer sequence	Reference	Size range (bp)
**Psol 3**	F: CACGACGTTGTAAAACGACCGGATCTTACCAGAATCAG R: GAGTAGCCTTTGGTTGTTGC	[35]	138–184.1
**Psol 6**	F: CACGACGTTGTAAAACGACCTCTTGGCAAAAGTCACCTG R: ATCCCATACCGATCTCCTTC		155–158.6
**Psol 10**	F: CACGACGTTGTAAAACGACAGACGGATTCCCACTGAT R: GGACTTGTAACCCATCGAGA		150–153.4

PCR products were analyzed using a 4300 DNA Analyzer (LI-COR, Lincoln, Nebraska, USA). Allele sizes were obtained by comparison with 50–700 bp standards (IRDye 700). Banding pattern analyses were performed using the GeneMapper software (Applied Biosystems) [26].

### Data analysis

#### Sequence quality evaluation

Sequence alignment was performed with the MUSCLE program [45], using default parameters and further manual optimization.

#### Identification test using local BLAST

A local BLAST search was used as a test of species identification ability [46]. A reference library was constructed using the "makeblastDB" command in BLAST for each region (ITS2, *trnH-psbA* and *rbcL*) and the combination ITS2+*trnH-psbA*. The BLASTn search method was used to test the species identification capacity, as previously described. This method was applied to the three markers and combinations between markers. A species sequence representing the genus was used as a query sequence in BLASTn searches. Species identification was based on sequences from the database with the highest index of identity, highest score, and lowest *e*-value.

#### Distance analysis

Intra and interspecific divergence patterns were estimated for the genomic regions ITS2, *trnH-psbA*, *rbcL* and combinations among them, based on the Kimura method 2 parameters (K2P), using the MEGA 6 software [47]. Wilcoxon signed rank tests were performed to compare interspecific variability for every barcode pair [10]. Barcoding gaps between interspecific and intraspecific distances were evaluated using frequency histograms based on the paired p-distances obtained with the Mega 6 software package [48].

#### Phylogenetic inference and cluster analysis

Phylogenetic inference based on DNA sequences and the Maximum Parsimony (MP) method was analyzed with MEGA 6 [48]. For analysis of each DNA region, the Subtree-Pruning-Regrafting (SPR) algorithm was chosen, saving the first 100 more parsimonious trees. Values of clade consistencies were obtained with the bootstrap (BS) method [49] with 1000 replicates. The reliability intervals of consistency of BS values were assumed as follows: 50–74—weak; 75–84—moderate; 85–100—strong [50]. For each analysis, the number of informative characters, consistency indexes (CI) and retention indexes (RI) were determined. As out groups, sequences from NCBI/Genebank of the following species were used: *rbcL*—*Piper longum* L. (KF432059.1); *trnH-psbA*—*P*. *betle* L. (JQ248053.1); ITS2 –*P*. *imperiale* (Miq.) C.DC. (EF056264). Distinct outgroups were used, because sequences of all three DNA regions for the same *Piper* species are not available in Genbank.

## Results

Amplification for DNA sequencing was successful for all individuals of *P*. *regnellii* and *P*. *gaudichaudianum*. However, only four individuals of *P*. *malacophyllum* provided amplified products (S1 Fig). Considering all *Piper* populations and the three DNA regions, a total of 96 sequences were obtained. All sequences generated in this study has been submitted to GenBank (S1 Table). Amplification products were obtained with SSR primers Psol 3, Psol 6 and Psol 10 for *P*. *gaudichaudianum* and *P*. *regnellii*, but not for *P*. *malacophyllum*.

### Success rates of PCR amplification, sequencing, and detection of SSR segments

The amplification rates were high regarding ITS2 (100%) and *rbcL* (90%) and moderate regarding *trnH-psbA* (70%). Sequencing success rates were high for *rbcL* and ITS2 (100% and 95%, respectively) and moderate for *trnH-psbA* (86%).

Capillary electrophoresis analysis detected SSR segments from 72% of the samples. The amplification of Psol 3, Psol 6 and Psol 10 was successful for *P*. *gaudichaudianum* and *P*. *regnellii* (97% rate), but not for *P*. *malacophyllum*. While amplification was successful for all populations of *P*. *gaudichaudianum* using primers of the three SSR markers, amplification failed for samples of *P*. *regnellii* from the USP populations using Psol 3 and Psol 6 primers. Table 3 contains data regarding length polymorphism of the observed microsatellite fragment sizes.

### Local BLASTn

The sequences of regions ITS2 and the sequence combination ITS2+*trnH-psbA* provided high identity (I) and score (S) values, as well as null *e*-values (Table 4).

**Table 4:** Results obtained from local blast by region.

Species	ITS2			ITS2*+trnH-psbA*		
	I	S	e	I	S	e
***P*. *regnellii***						
*P*. *regnellii* (Intervales) 3	98	775	0	98	1298	0
*P*. *regnellii* (Intervales) 4	98	791	0	98	1320	0
*P*. *regnellii* (Intervales) 5	98	779	0	98	1334	0
*P*. *regnellii* (Intervales) 10	98	791	0	-	-	-
*P*. *regnellii* (USP) 4	98	783	0	100	1427	0
*P*. *regnellii* (USP) 5	98	791	0	98	1348	0
*P*. *regnellii* (USP) 6	99	799	0	98	1298	0
*P*. *regnellii* (USP) 9	98	773	0	-	-	-
*P*. *regnellii* (USP) 10	100	839	0	98	1326	0
***P*. *gaudichaudianum***						
P. *gaudichaudianum* (Intervales) 3	100	829	0	99	1376	0
*P*. *gaudichaudianum* (Intervales) 4	100	829	0	98	1308	0
*P*. *gaudichaudianum* (Intervales) 5	100	829	0	99	1376	0
*P*. *gaudichaudianum* (Intervales) 6	100	829	0	98	1330	0
*P*. *gaudichaudianum* (Intervales) 7	99	821	0	98	1368	0
*P*. *gaudichaudianum* (Intervales) 8	100	842	0	98	1362	0
*P*. *gaudichaudianum* (USP) 1	100	616	0	-	-	-
*P*. *gaudichaudianum* (USP) 3	99	833	0	100	1437	0
*P*. *gaudichaudianum* (USP) 4	99	809	0	-	-	-
*P*. *gaudichaudianum* (USP) 7	100	829	0	99	1376	0
*P*. *gaudichaudianum* (USP) 8	100	829	0	98	1368	0
*P*. *gaudichaudianum* (USP) 9	99	817	0	99	1352	0
***P*. *malacophyllum***						
*P*. *malacophyllum* 1	100	841	0	100	1437	0
*P*. *malacophyllum* 2	100	841	0	99	1366	0

Abbreviations: I—Identity; S—Score; and *e*-value. (-) represent sequences not obtained in this study.

### Molecular divergence between and within species

**Fig 1:**
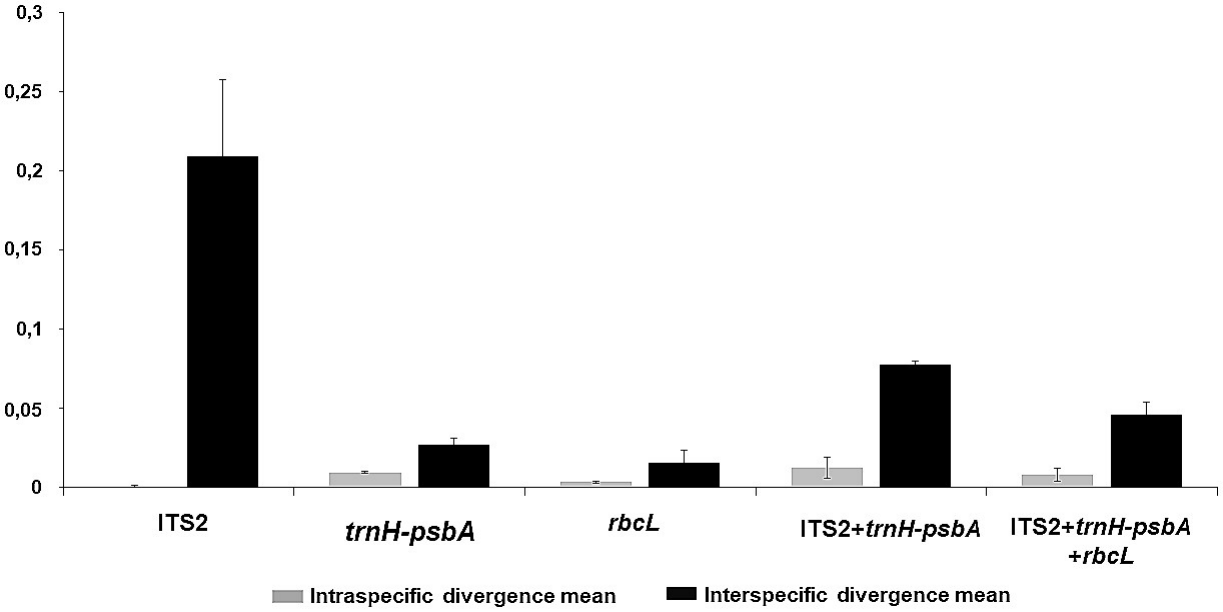
Estimate means of intraspecific and interspecific evolutionary diversities.

Means of interspecific genetic diversity varied from 0.02 (*rbcL*; *P*. *gaudichaudianum* x *P*. *malacophyllum*) to 0.21 (ITS2; *P*. *regnellii* x *P*. *malacophyllum*), while the diversity mean within population showed values from 0.0035 (*rbcL*) to 0.013 (ITS2+*trnH-psbA*) (Fig 1). Sequences of all three genomic regions showed levels of intraspecific genetic divergence lower than interspecific variation (Fig 1).

Wilcoxon signed rank tests confirmed that the internal transcribed spacer 2 (ITS2) provided the highest interspecific divergence, which was significantly higher than those of other regions. The second highest interspecific divergence corresponded to the *trnH-psbA* intergenic spacer, ITS2+*trnH-psbA* and ITS2+*trnH-psbA+rbcL* combined data, whereas *rbcL* showed the lowest divergence (Table 5).

**Table 5:** Wilcoxon signed rank tests of interspecific divergence among loci.

*Locus pairs*		*Relative ranks*	*P-value*	*Result*
W (+)	W (-)			
ITS2	*trnH-psbA*	W + = 36, W— = 264	p < = 0.001	ITS2 > *trnH-psbA*
ITS2	*rbcL*	W + = 0, W— = 300	p < = 0.001	ITS2 > *rbcL*
ITS2	ITS2+*trnH-psbA*	W + = 0, W— = 171	p < = 0.001	ITS2 > ITS2+*trnH-psbA*
ITS2	ITS2+*trnH*-*psbA*+*rbcL*	W + = 0, W— = 231	p < = 0.001	ITS2 > ITS2+*trnH-psbA+rbcL*
*trnH-psbA*	*rbcL*	W + = 0, W— = 300	p < = 0.001	*trnH-psbA > rbcL*
*trnH-psbA*	ITS2+*trnH-psbA*	W + = 203, W— = 28	p < = 0.002	*trnH-psbA >* ITS2+*trnH-psbA*
*trnH-psbA*	ITS2+*trnH-psbA+rbcL*	W + = 203, W— = 28	p < = 0.002	*trnH-psbA<*ITS2+*trnH-psbA+rbcL*
*rbcL*	ITS2+*trnH-psbA*	W + = 203, W— = 28	p < = 0.002	*rbcL <* ITS2+*trnH-psbA*
*rbcL*	ITS2+*trnH-psbA+rbcL*	W + = 231, W— = 0	p < = 0.001	*rbcL <* ITS2+*trnH-psbA+rbcL*

**Fig 2:**
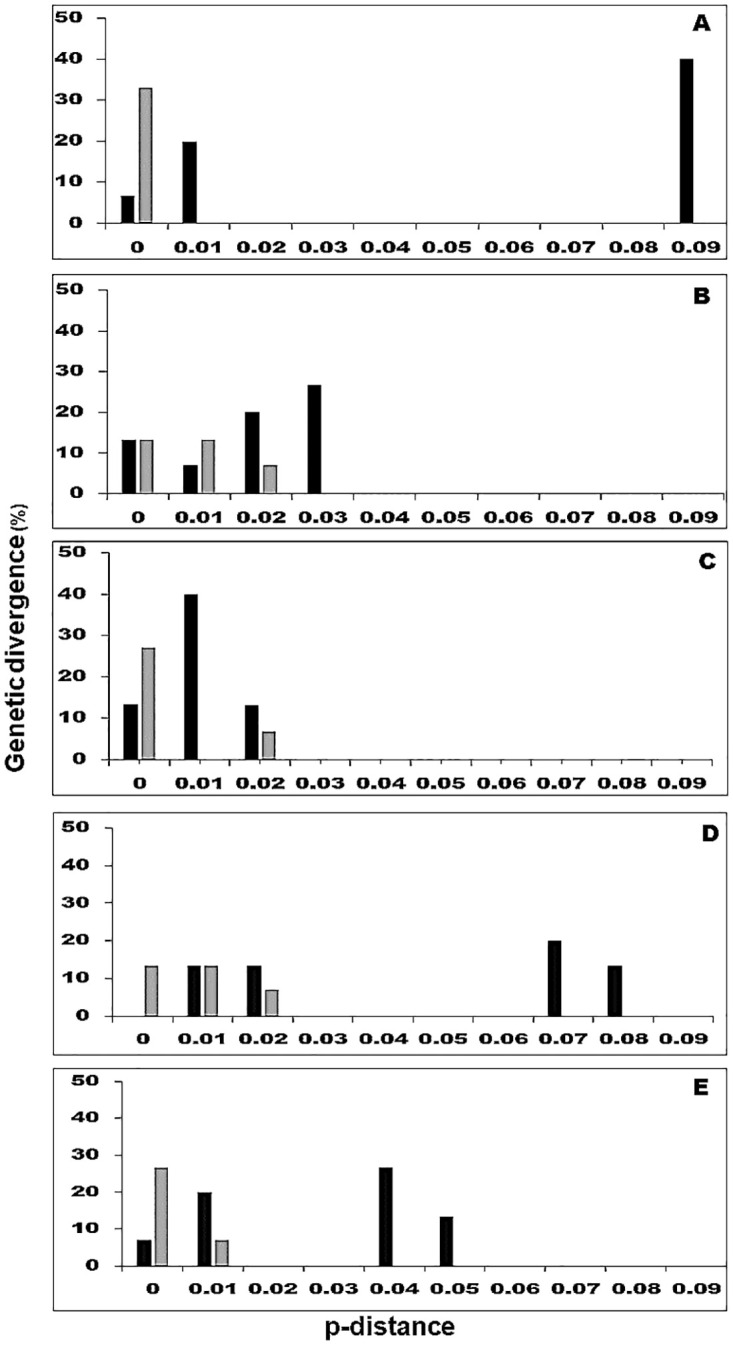
Histograms of the frequencies (y-axes) of pairwise intraspecific (grey bars) and interspecific (black bars) divergences based on the p-distance (x-axes) for individual and combined ITS2, *trnH-psbA*, and *rbcL* markers for all species. A: ITS2; B: *trnH-psbA*; C: *rbcL*; D: ITS2+*trnH-psbA*; E: ITS2+*trnH-psbA+rbcL*.

Barcodes should generally demonstrate a “barcoding gap” between intra- and interspecific distances. To evaluate the gaps, we distributed the divergences in classes of 0.01 distance units for all species (Fig 2) and each species (S2 and S3 Figs).

### Phylogenetic analyses

**Fig 3:**
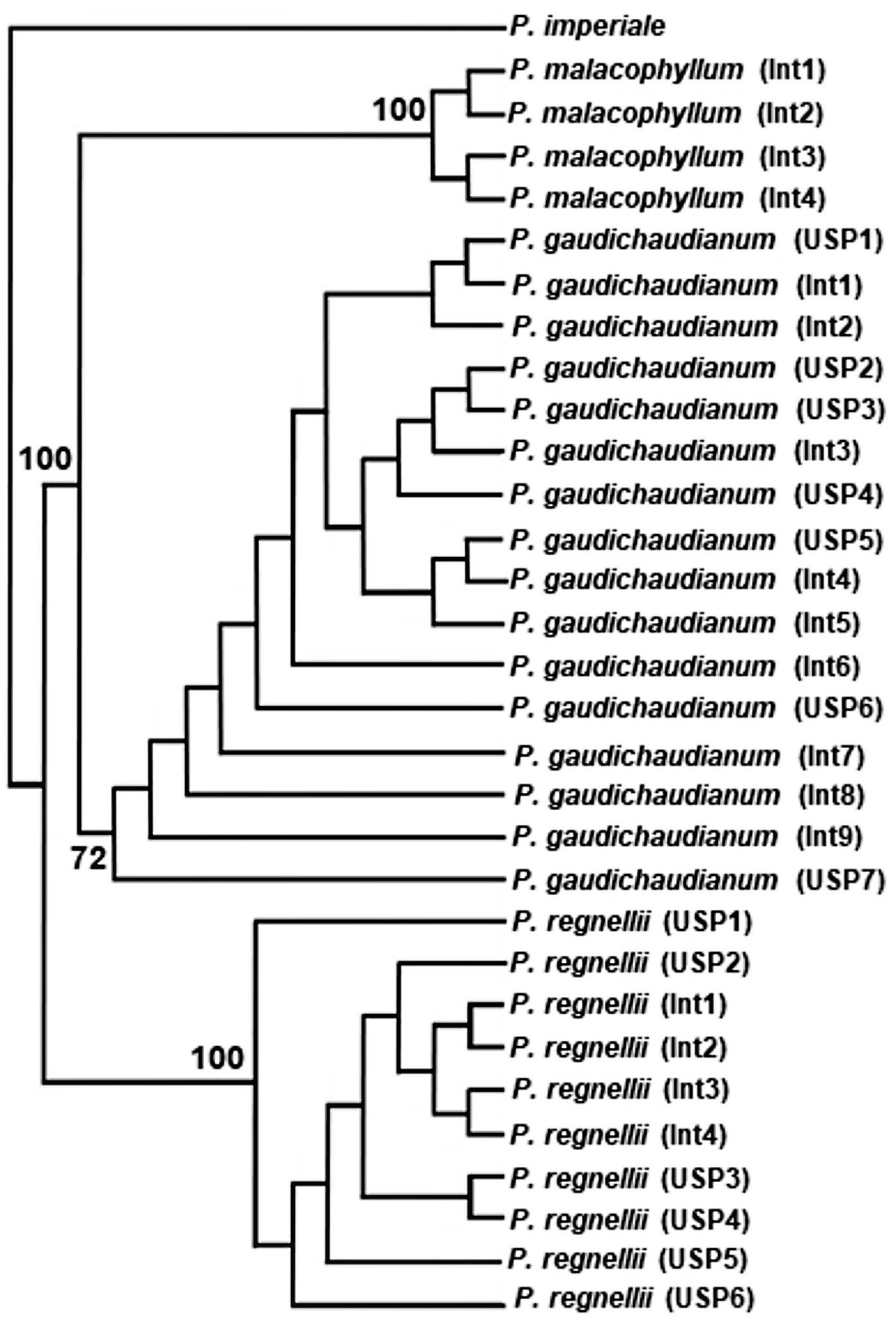
Strict consensus trees based on maximum parsimony of ITS2 sequences of *Piper* species. Bootstrap support values above 50% are shown above branches. USP, Int: population of *Piper* species from University of São Paulo and Intervales State Park (Ribeirão Grande, São Paulo State), respectively.

**Fig 4:**
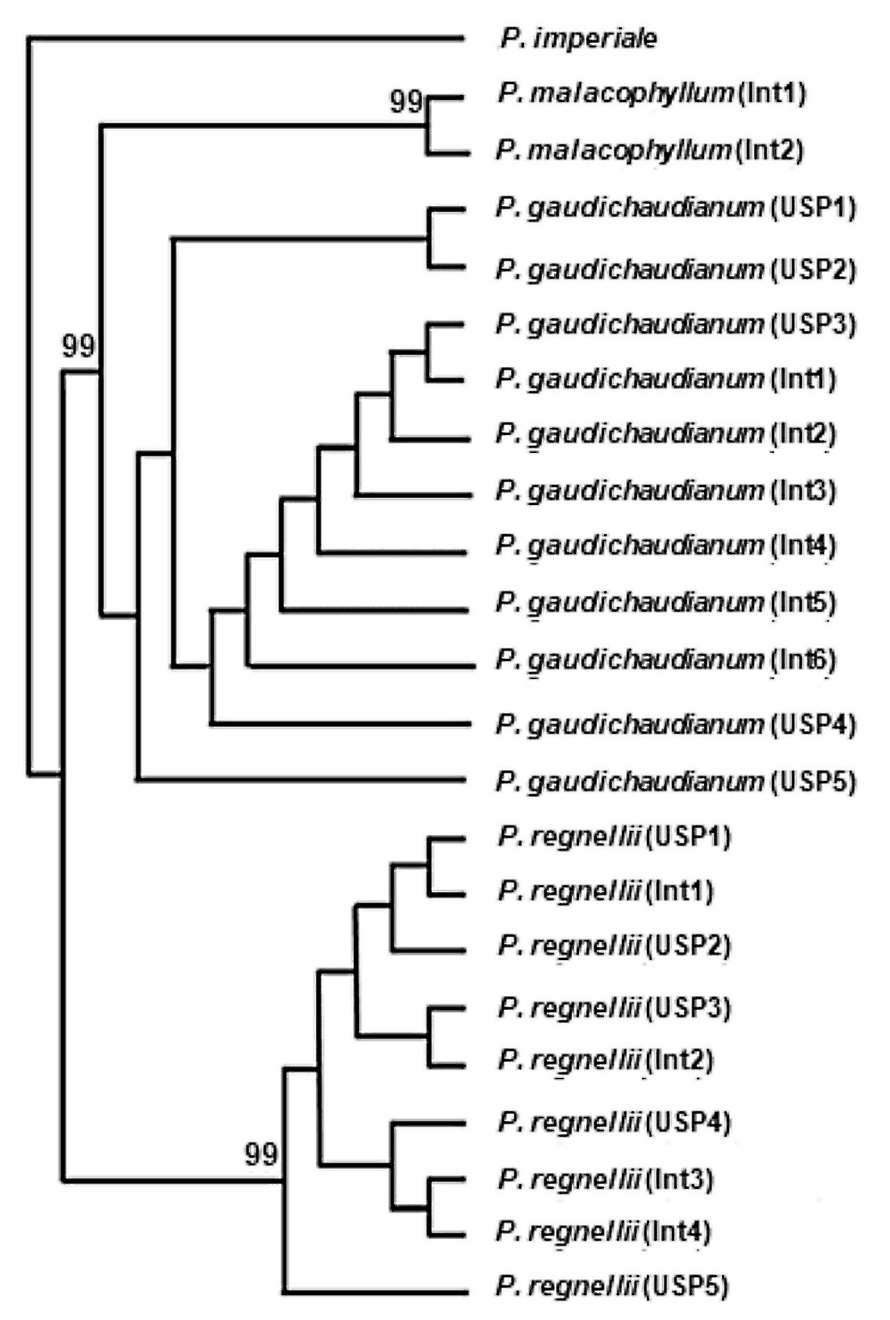
Strict consensus trees based on maximum parsimony of combined sequences of ITS2+*trnH-psbA* from *Piper* species. Bootstrap support values above 50% are shown above branches. USP, Int: population of *Piper* species from University o0066 São Paulo and Intervales State Park (Ribeirão Grande, São Paulo State), respectively.

Strict consensus trees from phylogenetic analyses of *Piper* populations, based on Maximum Parsimony analysis of sequences from the three DNA regions, are shown on Figs 3 and 4, S4–S7 Figs. Matrix parameters and details of the phylogenetic analyses are given in Table 6.

**Table 6:** Parameters of matrices and maximum parsimony phylogenetic analyses.

*Region*	*Maximum Parsimony*					*Fragment*
	Characters	terminals	steps	CI	RI	size (bp)
**ITS2**	420	31	210	0.971	0.982	560–580
***trnH-psbA***	270	27	118	0.966	0.916	300–310
***rbcL***	573	38	260	0.973	0.919	560–580
**ITS2*+trnH-psbA***	715	23	406	0.955	0.952	860–880
***trnH-psbA+rbcL***	862	27	417	0.953	0.836	860–870
**ITS2*+trnH-psbA+rbcL***	1286	23	416	0.956	0.954	1420–1560

*CI—Consistency index; RI—Retention index.

The tree based on ITS2 sequences (Fig 3) provided three clades: 1) *P*. *malacophyllum*, with high BootStrap value (100); 2) *P*. *gaudichaudianum*, weakly supported (BS 72); 3) *P*. *regnellii*, with robust BS (100). Sequences of *trnH-psbA* are useful for characterizing *P*. *regnellii* (BS 100), but not *P*. *gaudichaudianum* which was resolved as paraphyletic (S4 Fig). The DNA marker *trnH-psbA* might also be useful for *P*. *malacophyllum*, but the sample size was too small for a robust test. Sequences of *rbcL* are useful for characterization of *P*. *regnellii*, which claded with strong BS (100) (S5 Fig). However, no separation was possible between samples of *P*. *gaudichaudianum* and *P*. *malacophyllum*. A strongly supported clade (BS 100) comprising three individuals of *P*. *malacophyllum* is nested within populations of *P*. *gaudichaudianum*. The *P*. *gaudichaudianum*/*P*. *malacophyllum* clade is moderately supported (BS 80; S5 Fig).

Results resembling those obtained with sequences of *rbcL* were achieved with the combination ITS2+*trnH-psbA*, namely two strongly supported clades with one corresponding to *P*. *gaudichaudianum*/*P*. *malacophyllum* and the other *P*. *regnellii* (Fig 4). The combination *trnH-psbA*+*rbcL* was not able to successfully characterize of any of the three *Piper* species. Populations of *P*. *malacophyllum* emerged as a grade at the base of the tree, the clade joining all populations of *P*. *regnellii* has no BS support and *P*. *gaudichaudianum* is polyphyletic (S6 Fig). The combination ITS2+*trnH-psbA*+*rbcL* provided two polytomous clades: a strongly supported clade *P*. *gaudichaudianum/P*. *malacophyllum* (BS 100) and a moderately supported clade *P*. *regnellii* (BS 75) (S7 Fig).

**Fig 5:**
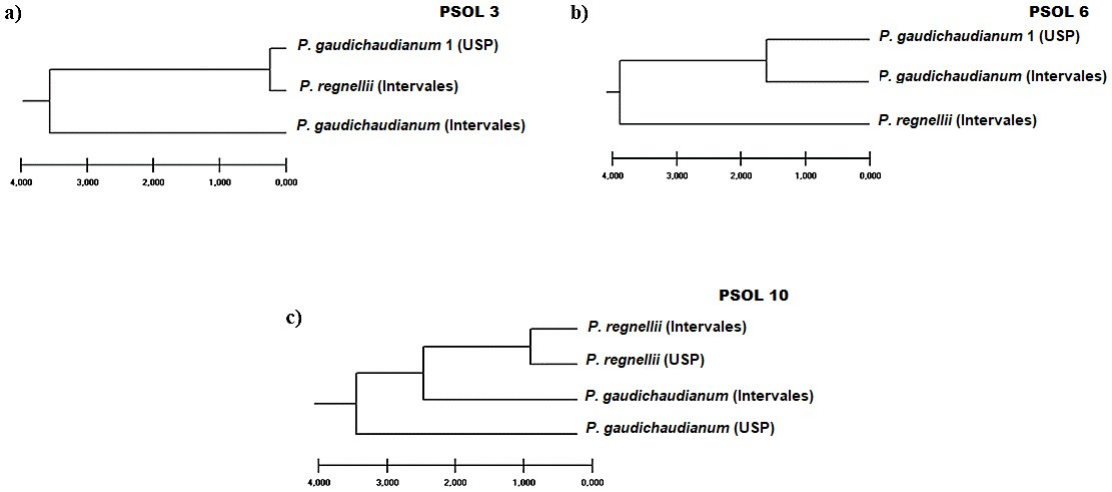
Affinity relationships among species and populations of *Piper* species, based on UPGMA clustering method and polymorphisms of amplification products derived from SSR markers Psol 3 (a), Psol 6 (b) and Psol 10 (c). USP, Int: population of *Piper* species from University of São Paulo and Intervales State Park (Ribeirão Grande, São Paulo State), respectively.

Cladograms obtained with data from Psol 3 and Psol 6, derived from the same populations, have distinct topologies (Fig 5a and 5b). Analysis based on Psol 3 failed to cluster the two samples of *P*. *gaudichaudianum* (Fig 5a). On the contrary, the same samples comprise a cluster on the cladogram based on Psol 6 (Fig 5b). The cladogram based on Psol 10 contains a cluster with the two samples of *P*. *regnellii*. On the other hand, the samples of *P*. *gaudichaudianum* do not cluster, but form a basal grade.

## Discussion

Efficiency of PCR amplification and sequencing is vital for evaluating molecular markers in DNA barcoding [18]. In this respect, the data presented in Table 3 are encouraging regarding the SSR markers and the three DNA regions evaluated here.

As a first evaluation of the sequences of ITS2, *rbcL* and *trnH*-*psbA* for barcoding within the genus *Piper*, the tests of local BLASTn provided promising results using both ITS2 alone, and the combination of ITS2+*trnH*-*psbA* (Table 4).

High variation between species and low variation within species is a fundamental requisite for genomic makers used for barcoding that are aimed a species identification and delimitation [15, 22]. Variation within all the DNA regions examined in this study were higher between species than within species (Fig 1). When individual and combined loci were analyzed, ITS2 presented the best barcode gap performance, with 40% of pairwise interspecific p-distances greater than 0.09 and 33% of pairwise intraspecific p-distances lower than 0.007. The second highest barcode gap performance resulted from the combination of ITS2+*trnH-psbA* and ITS2+*trnH-psbA+rbcL*. Conversely, *trnH-psbA and rbcL* showed the worst performance, with an overlap of intra- and interspecific variation (Fig 2). Analyses of the DNA barcoding gap and Wilcoxon two-sample tests support the notion that the mean interspecific divergence of the ITS2 is significantly higher than its mean intraspecific variation (Table 5).

The capacity of DNA sequences to clade populations of *Piper* species in phylogenetic analyses varied from one genomic region to another (Figs 3 and 4, S4–S7 Figs). ITS2 sequences enabled the delimitations of the three species, although the *P*. *gaudichaudianum* clade was weakly supported. Analyses based on the DNA regions *trnH-psbA* and *rbcL* were successful at grouping populations of *P*. *regnellii* into strongly supported clades, but not *P*. *gaudichaudianum* (S4 and S5 Figs). *ITS* was previously used in a large phylogenetic analysis including *Piper* species from around the world [51], but none of the three species examined here were included in that analysis. In this global phylogeny, ten major clades were recognized, and a morphological description was included for each. Some of these clades correspond to previously recognized sections, such as *Radula* and *Schilleria* [51]. A recent phylogenetic analysis including many species native to Brazil places *P*. *gaudichaudianum* and *P*. *malacophyllum* in section *Radula*, while *P*. *regnellii* belongs to section *Schilleria* (unpublished results). The close relationship between *P*. *gaudichaudianum* and *P*. *malacophyllum* may account for the difficulty at attaining separate and strongly supported clades for these two species using sequences of some genomic regions, such as *rbcL* (S5 Fig). The phylogeny based on *trnH-psbA* suggests the possibility that this marker might be a useful barcode for *P*. *malacophyllum* (S4 Fig); however, only a small subset of the samples included in this study amplified and sequenced successfully so the sample size presented here is too small to draw a definite conclusion. Although the combination of sequences from several genomic regions often improves resolution and clade support in phylogenetic analysis [52], our results indicate that no gain of resolution or BS support is attained with the combination of sequences of the DNA regions used in this study (Fig 4 and S7 Fig).

Transferability of the SSR markers using Psol primers were successful for *P*. *gaudichaudianum* and *P*. *regnellii*, but no amplified SSR segments were obtained from *P*. *malacophyllum*. SSR markers have been useful for evaluation of genetic diversity and establishment of affinity relationships among species of *Piper*, including *P*. *solmsianum* [35], *P*. *nigrum* [53] and *P*. *polysyphonum* C. DC. [54]. Results of preliminary cluster analysis based on polymorphisms of amplified products obtained with transferred SSR markers are shown on Fig 5a–5c. The number of polymorphic characters obtained is still too small to be confidently used as barcodes, which precludes any conclusion about the possibility of distinguishing the three species based on the transferred SSR markers. However, comparing the topologies of the dendrograms obtained, it appears that Psol 6 and Psol 10 (Fig 5b and 5c, respectively) may hold more promise for distinguishing *Piper* species than Psol 3 (Fig 5a). Further study is necessary to thorough evaluate Psol primers for delimiting and distinguishing among *Piper* species.

The phytochemistry of species *P*. *gaudichaudianum*, *P*. *malacophyllum* and *P*. *regnellii* have been investigated. While *P*. *gaudichaudianum* was characterized by the presence of prenylated benzoic acids such as gaudichaudianic acid [55] and taboganic acid [56], *P*. *malacophyllum* was shown to contain piperolides, a specific type of polyketide [6]. The chemistry of *P*. *regnellii* was shown to contain dihydrobenzofuran neolignans exemplified as conocarpan, eupomatenoids and phenylpropanoids [57–58]. The close phylogenetic relationship between *P*. *gaudichaudianum* and *P*. *malacophyllum* is not reflected in the secondary chemical profiles of these species.

The molecular markers used in the present study may be useful for a variety of biological and economic applications, such as species identification, even in the absence of flowers and fruits, which are necessary for accurate identification of many *Piper* species [59–60], authentication and quality control of medicinal plants [14], as well as quality control in analyses of food safety [32], as support for studies of chemical variability and for accurate understanding of ecological relationships [61]. Thus, the development of tools to accurately identify species in the large, complicated, and economically important genus *Piper* is of great interest.

## Conclusions

Sequences of ITS2 enabled delimitation of the three *Piper* species and provided a high degree of intraspecific stability. Analyses based on *trnH-psbA*, *rbcL* and combination of both sequences were successful at grouping populations of *P*. *regnellii*, but not of *P*. *gaudichaudianum*. Transferability of SSR Psol primers is feasible for *P*. *gaudichaudianum* and *P*. *regnellii*. Preliminary analysis suggests that barcoding of *Piper* species based on polymorphisms of Psol microsatellites holds promise.

## Supporting information

S1 Fig Raw gel electrophoresis photograph of DNA and PCR products in the ITS2, *trnH-psbA* and *rbcL* region detected by 2% agarose. LaneM: 1Kb Plus DNA Ladder; Lane1-19: representative samples of *P*. *gaudichaudianum*, *P*. *malacophyllum* and *P*. *regnellii*.
(TIF)

S2 Fig Histograms of frequencies (y-axes) of pairwise intraspecific (grey bars) and interspecific (black bars) divergences based on p-distances (x-axes) for individual and combined ITS2, *trnH-psbA*, and *rbcL* markers for *P*. *regnellii*. A: ITS2; B: *trnH-psbA*; C: *rbcL*; D: ITS2+*trnH-psbA*; E: ITS2+*trnH-psbA+rbcL*.
(TIF)

S3 Fig Histograms of frequencies (y-axes) of pairwise intraspecific (grey bars) and interspecific (black bars) divergences based on p-distances (x-axes) for individual and combined ITS2, *trnH-psbA*, and *rbcL* markers for *P*. *gaudichaudianum*. A: ITS2; B: *trnH-psbA*; C: *rbcL*; D: ITS2+*trnH-psbA*; E: ITS2+*trnH-psbA+rbcL*.
(TIF)

S4 Fig Strict consensus tree based on sequences of *trnH*-*psbA* of *Piper* species, based on maximum parsimony. Bootstrap support values above 50% are shown above branches. USP, Int: population of *Piper* species from University of São Paulo and Intervales State Park (Ribeirão Grande, São Paulo State), respectively.
(TIF)

S5 Fig Strict consensus tree based on sequences of *rbcL* of *Piper* species, based on maximum parsimony. Bootstrap support values above 50% are shown above branches. USP, Int: population of *Piper* species from University of São Paulo and Intervales State Park (Ribeirão Grande, São Paulo State), respectively.
(TIF)

S6 Fig Strict consensus trees based on combined sequences of *trnH-psbA*+*rbcL* of *Piper* species, based on maximum parsimony. Bootstrap support values above 50% are shown above branches. USP, Int: population of *Piper* species from University of São Paulo and Intervales State Park (Ribeirão Grande, São Paulo State), respectively.
(TIF)

S7 Fig Strict consensus trees based on combined sequences of ITS2+*trnH*-*psbA*+rbcL of *Piper* species, based on maximum parsimony. Bootstrap support values above 50% are shown above branches. USP, Int: population of *Piper* species from University of São Paulo and Intervales State Park (Ribeirão Grande, São Paulo State), respectively.
(TIF)

S1 Table International nucleotide sequence database collaboration GenBank. (-) Indicates no sequence obtained.
(DOCX)
