# Supplementary figures and images for: Evaluation of DNA markers for molecular identification of three Piper species from Brazilian Atlantic Rainforest
Source: PLoS One. 2020 Oct 19;15(10):e0239056. doi: 10.1371/journal.pone.0239056 (PMC7571689; doi:10.1371/journal.pone.0239056)

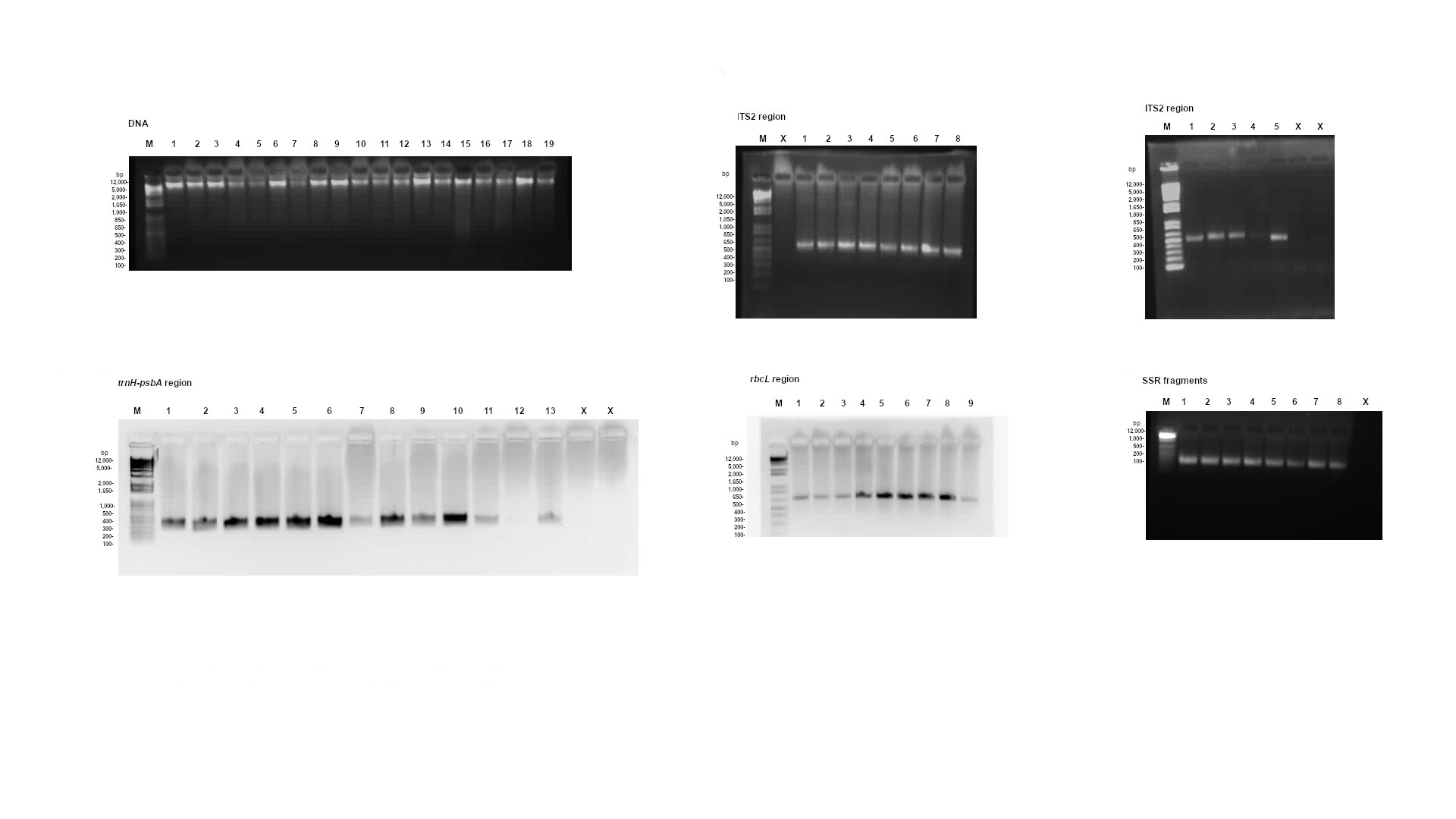

Supplement: S1 Fig — LaneM: 1Kb Plus DNA Ladder; Lane1-19: representative samples of P. gaudichaudianum, P. malacophyllum and P. regnellii. (TIF) [file pone.0239056.s001.tif]

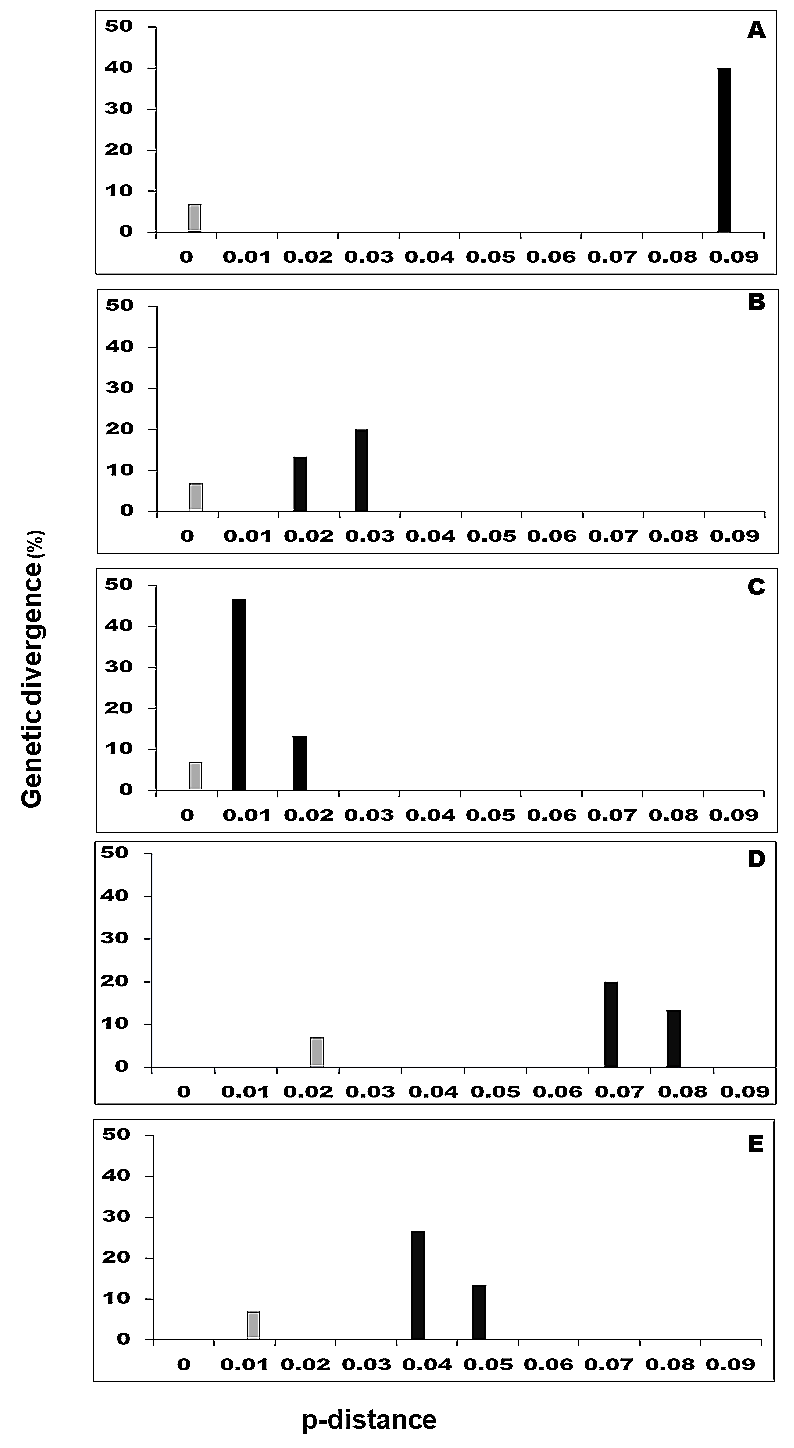

Supplement: S2 Fig — A: ITS2; B: trnH-psbA; C: rbcL; D: ITS2+trnH-psbA; E: ITS2+trnH-psbA+rbcL. (TIF) [file pone.0239056.s002.tif]

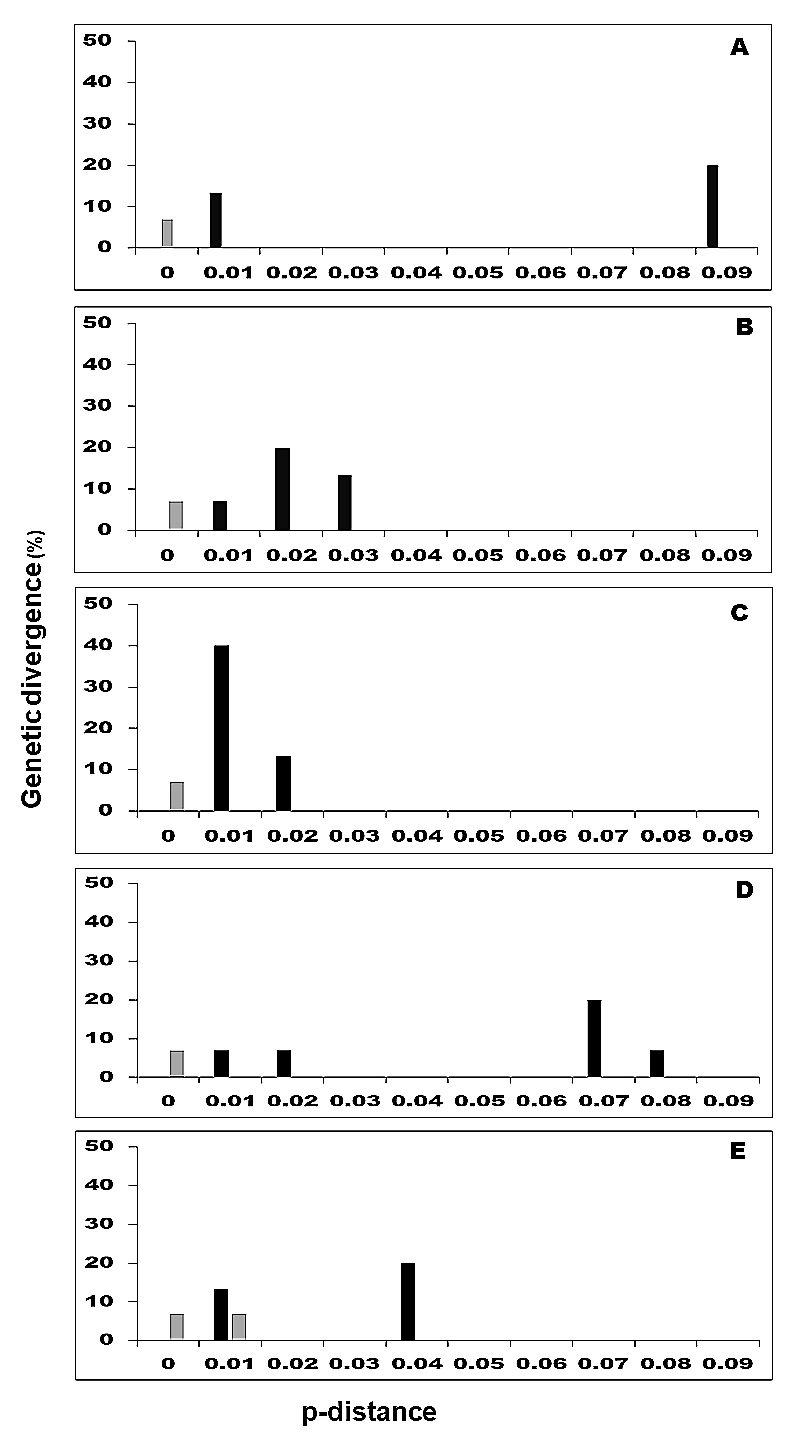

Supplement: S3 Fig — A: ITS2; B: trnH-psbA; C: rbcL; D: ITS2+trnH-psbA; E: ITS2+trnH-psbA+rbcL. (TIF) [file pone.0239056.s003.tif]

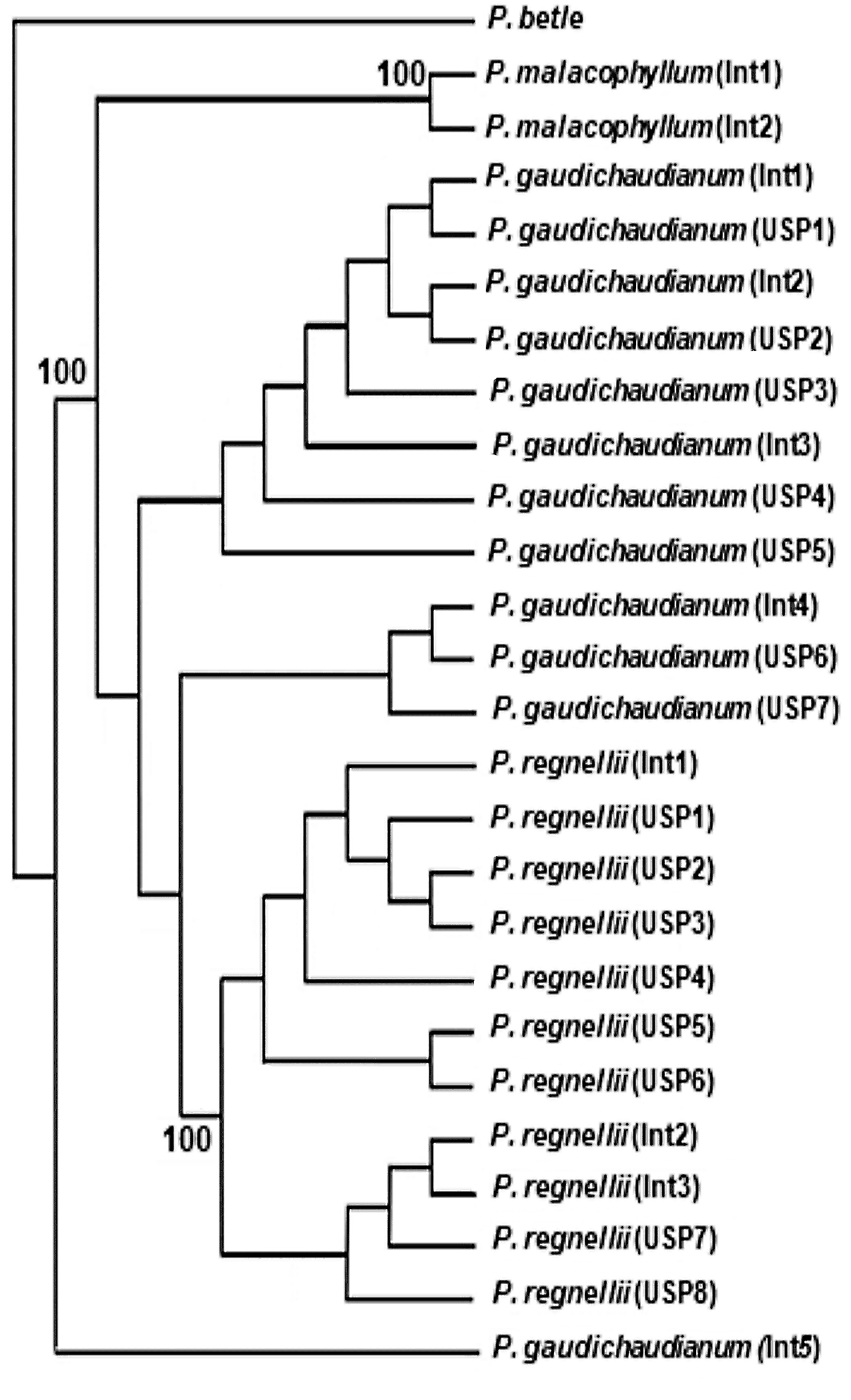

Supplement: S4 Fig — Bootstrap support values above 50% are shown above branches. USP, Int: population of Piper species from University of São Paulo and Intervales State Park (Ribeirão Grande, São Paulo State), respectively. (TIF) [file pone.0239056.s004.tif]

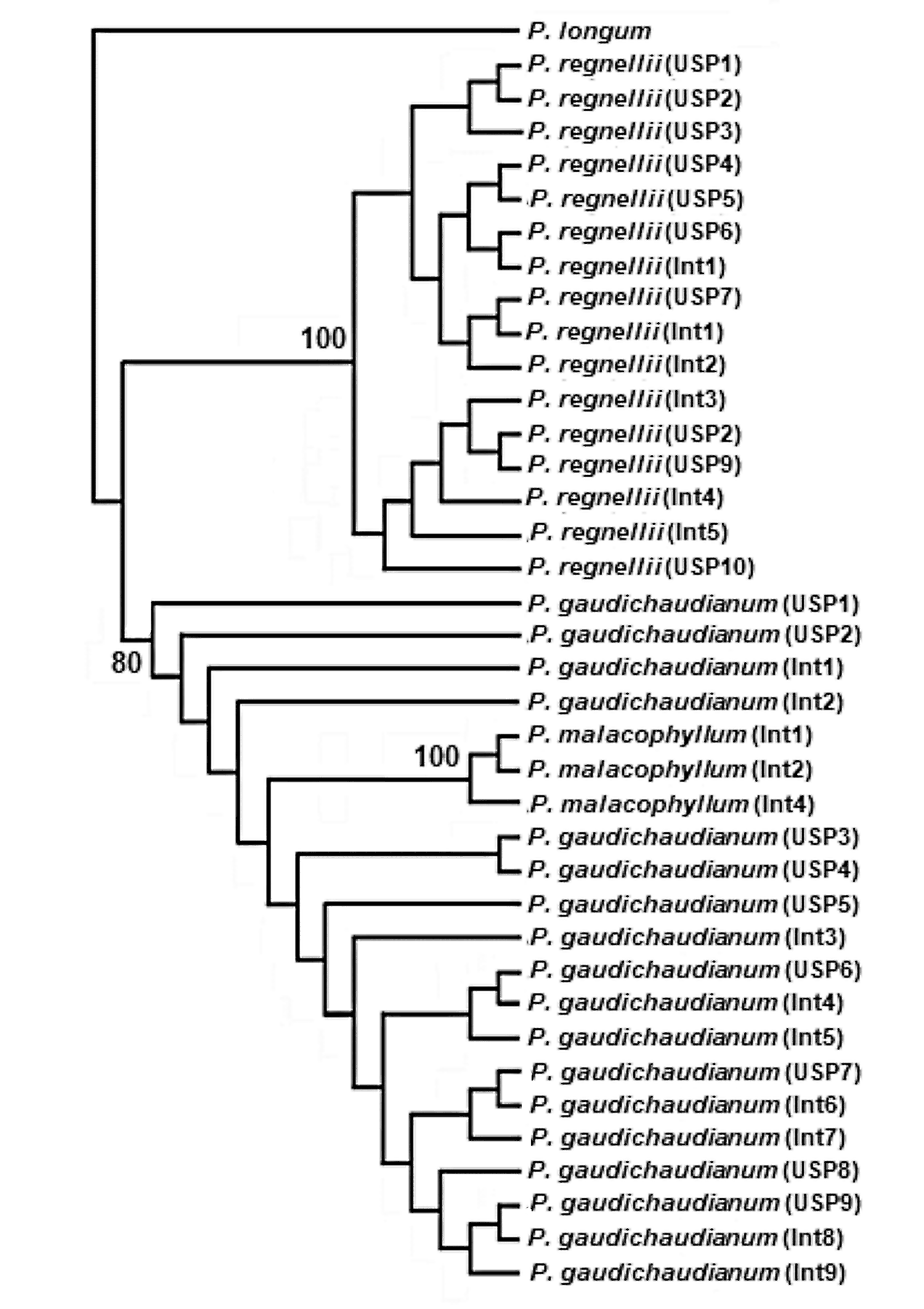

Supplement: S5 Fig — Bootstrap support values above 50% are shown above branches. USP, Int: population of Piper species from University of São Paulo and Intervales State Park (Ribeirão Grande, São Paulo State), respectively. (TIF) [file pone.0239056.s005.tif]

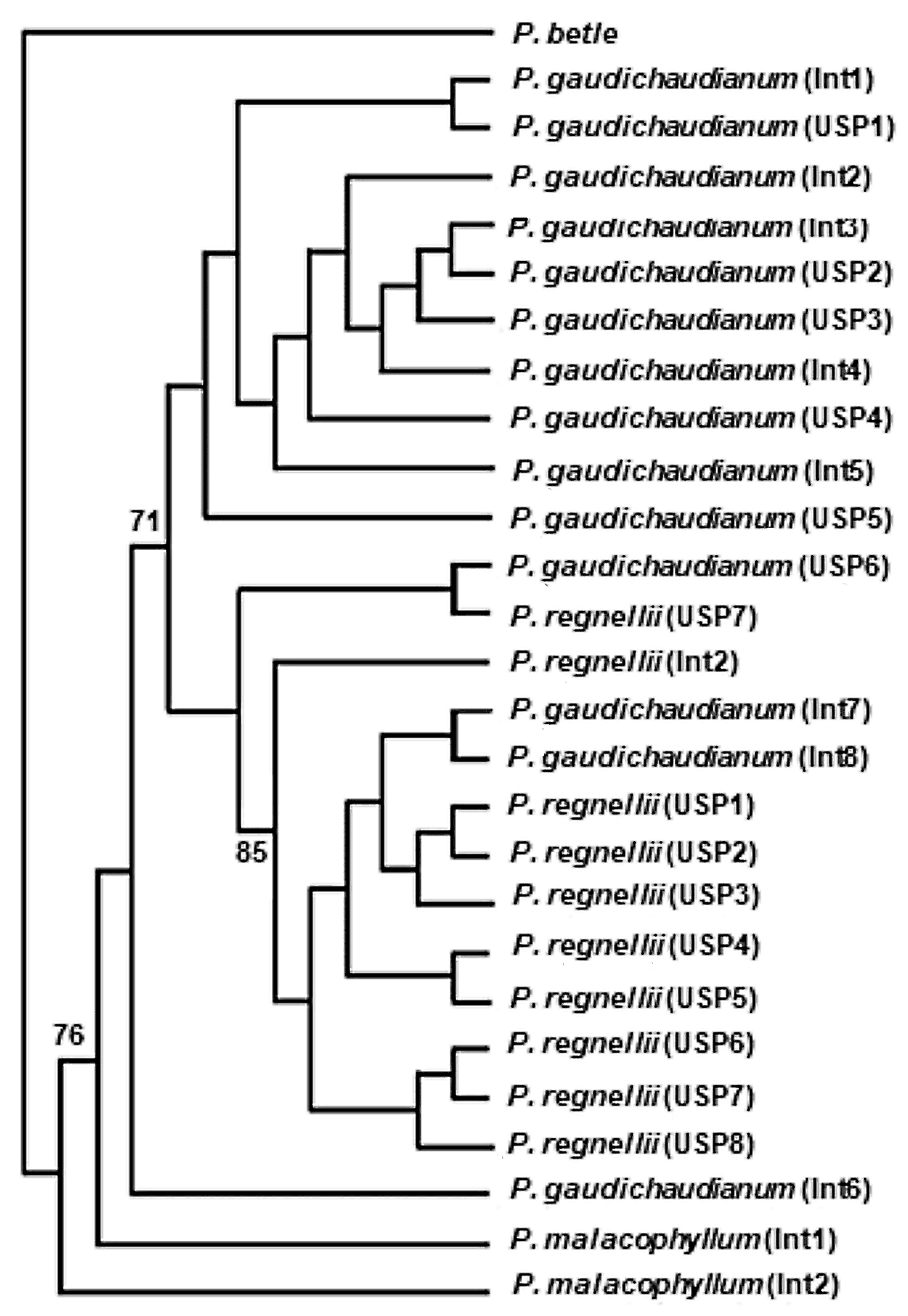

Supplement: S6 Fig — Bootstrap support values above 50% are shown above branches. USP, Int: population of Piper species from University of São Paulo and Intervales State Park (Ribeirão Grande, São Paulo State), respectively. (TIF) [file pone.0239056.s006.tif]

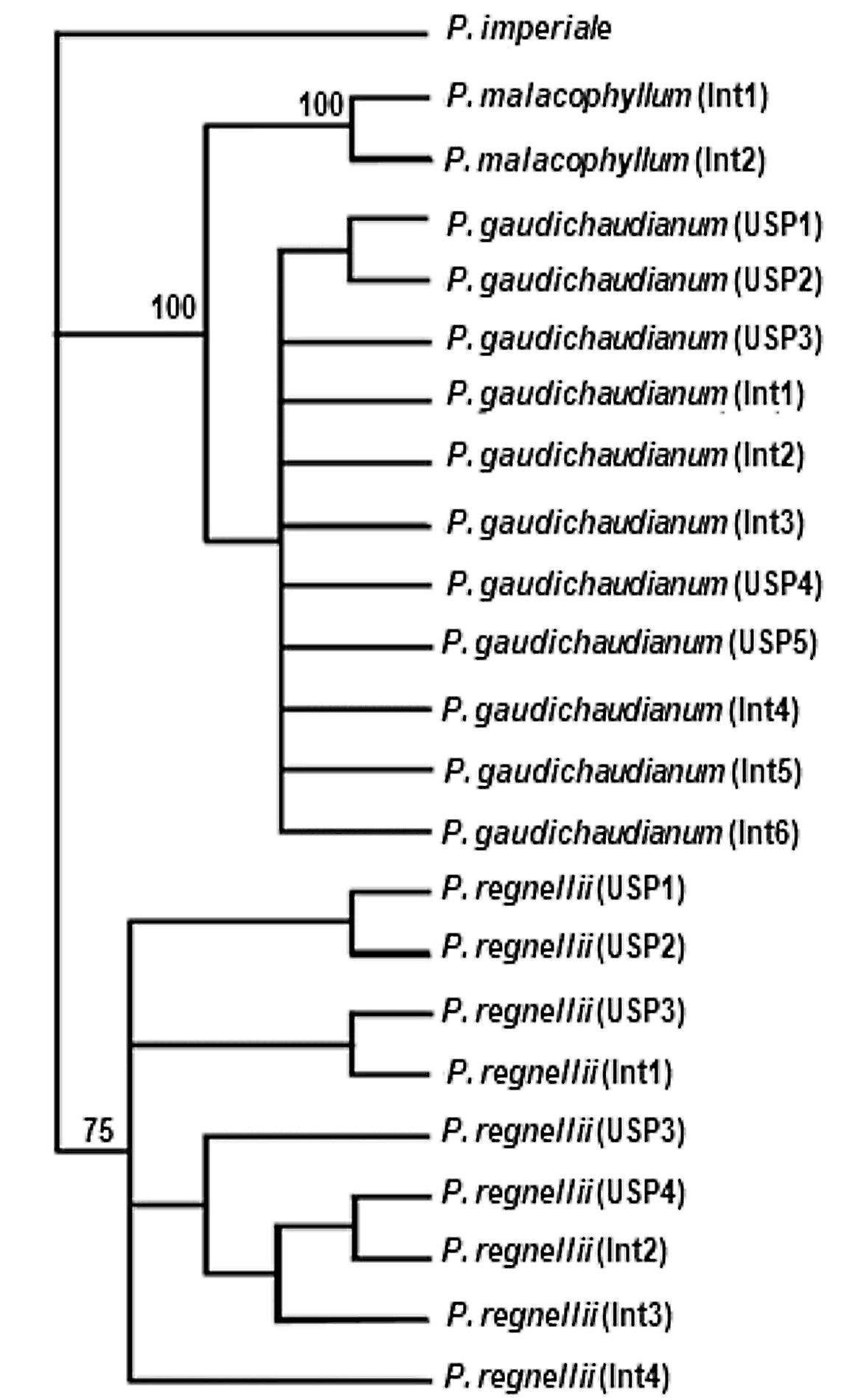

Supplement: S7 Fig — Bootstrap support values above 50% are shown above branches. USP, Int: population of Piper species from University of São Paulo and Intervales State Park (Ribeirão Grande, São Paulo State), respectively. (TIF) [file pone.0239056.s007.tif]
