# Supplementary material for: Evaluation of DNA markers for molecular identification of three Piper species from Brazilian Atlantic Rainforest
Source: PLoS One. 2020 Oct 19;15(10):e0239056. doi: 10.1371/journal.pone.0239056 (PMC7571689; doi:10.1371/journal.pone.0239056)
Supplement: S1 Table — (-) Indicates no sequence obtained. (DOCX) [file pone.0239056.s008.docx]

**S1 Table - International Nucleotide Sequence Database Collaboration GenBank.**

| ***Species*** | ***Voucher specimen*** | ***GenBank access number***  ***(GenBank deposit)*** | | | | |  |
| --- | --- | --- | --- | --- | --- | --- | --- |
|  |  | ***trnH-psbA*** | | | ***rbcL*** | **ITS2** | |
| *P. gaudichaudianum*  Kunth . | M. Kato K-031 | MT295798  (BankIt2330284) | | | MT530299  (BankIt2348773) | MT279554  (SUB7245207) | |
|  |  | MT295799  (BankIt2330284) | | | MT530300  (BankIt2348773) | MT279555  (SUB7245207) | |
|  |  | MT295800  (BankIt2330284) | | | MT530301  (BankIt2348773) | MT279556  (SUB7245207) | |
|  |  | MT295801  (BankIt2330284) | | | MT530302  (BankIt2348773) | MT279557  (SUB7245207) | |
|  |  | MT295802  (BankIt2330284) | | | MT530303  (BankIt2348773) | MT279558  (SUB7245207) | |
|  |  | - | | | MT530304  (BankIt2348773) | MT279559  (SUB7245207) | |
|  |  | - | | | MT530297  (BankIt2348757) | MT279560  (SUB7245207) | |
|  |  | -  - | | | MT530296  (BankIt2348753) MT530295  (BankIt2348750) | -  - | |
|  | M. Kato K-1983 | MT295790  (BankIt2330284) | | | MT530305  (BankIt2348807) | MT279561  (SUB7245207) | |
|  |  | MT295791  (BankIt2330284) | | | MT530306  (BankIt2348807) | MT279562  (SUB7245207) | |
|  |  | MT295792  (BankIt2330284) | | | MT530307  (BankIt2348807) | MT279563  (SUB7245207) | |
|  |  | MT295793  (BankIt2330284) | | | MT530308  (BankIt2348807) | MT279564  (SUB7245207) | |
|  |  | MT295794  (BankIt2330284) | | | MT530309  (BankIt2348807) | MT279565  (SUB7245207) | |
|  |  | MT295795  (BankIt2330284) | | | MT530310  (BankIt2348807) | MT279566  (SUB7245207) | |
|  |  | MT295796  (BankIt2330284) | | | MT530311  (BankIt2348807) | MT279567  (SUB7245207) | |
|  |  | MT295797  (BankIt2330284) | | | MT530312  (BankIt2348807) | MT279568  (SUB7245207) | |
|  |  | - | | | MT530298  (BankIt2348767) | MT279569  (SUB7245207) | |
| *P. malacophyllum* (C.Presl) C.DC. | M. Kato K-448 | MT295788  (BankIt2329035) | | | MT530279  (BankIt2347922) | MT293598  (SUB7245039) | |
|  |  | MT295789  (BankIt2329035) | | | MT530313  (BankIt2349468) | MT293599  (SUB7245039) | |
|  |  | - | | | MT492010 (BankIt23467749) | MT293600  (SUB7245039) | |
|  |  | - | | | - | MT755619  (SUB7758080) | |
| *P. regnellii* (Miq.) C.DC.  continued | M. Kato K-1452 | MT295803  (BankIt2330565) | | | MT530291  (BankIt2348746) | MT279541  (SUB7245185) | |
|  |  | MT295804  (BankIt2330565) | | | MT530292  (BankIt2348746) | MT279542  (SUB7245185) | |
|  |  | MT755644  (BankIt2364997) | | | MT530293  (BankIt2348746) | MT279543  (SUB7245185) | |
|  |  | - | | | MT530294  (BankIt2348746) | MT279544  (SUB7245185) | |
|  |  | - | | | MT530286  (BankIt2348739) | - | |
|  |  |  | | |  |  | |
|  | M. Kato K-242 | MT295805  (BankIt2330565) | | | MT530289  (BankIt2348744) | MT279545  (SUB7245185) | |
|  |  | MT295806  (BankIt2330565) | | | MT530290  (BankIt2348744) | MT279546  (SUB7245185) | |
|  |  | MT295807  (BankIt2330565) | | | MT530288  (BankIt2348741) | MT279547  (SUB7245185) | |
|  |  | MT295808  (BankIt2330565) | | | MT530287  (BankIt2348740) | MT279548  (SUB7245185) | |
|  |  | MT295809  (BankIt2330565) | | | MT530285  (BankIt2348736) | MT279549  (SUB7245185) | |
|  |  | MT295810  (BankIt2330565) | | | MT530284  (BankIt2348732) | MT279550  (SUB7245185) | |
|  |  | MT295811 (BankIt2330565) | | | MT530283  (BankIt2348729) | - | |
|  |  | MT295812  (BankIt2330565) | | MT530282 (BankIt2348727) | | - |  |
|  |  | - | | MT530314  (BankIt2349477) | | - |  |
|  |  | | - | MT530281  (BankIt2348692 | | - |  |

(-) Indicates no sequence obtained.
